# Plant Growth in LED-Sourced Biophilic Environments Is Improved by the Biochar Amendment of Low-Fertility Soil, the Reflection of Low-Intensity Light, and a Continuous Photoperiod

**DOI:** 10.3390/plants12183319

**Published:** 2023-09-20

**Authors:** Peter Beatrice, Alessio Miali, Silvia Baronti, Donato Chiatante, Antonio Montagnoli

**Affiliations:** 1 Department of Biotechnology and Life Sciences, University of Insubria, 21100 Varese, Italy; a.miali@studenti.uninsubria.it (A.M.); donato.chiatante@uninsubria.it (D.C.); antonio.montagnoli@uninsubria.it (A.M.); 2 Institute of BioEconomy, National Research Council, 50145 Firenze, Italy; silvia.baronti@ibe.cnr.it

**Keywords:** CoeLux^®^, light-emitting diode, *Arabidopsis thaliana*, photomorphogenesis, confined environment, low light intensity, biophilia, peat, mirror, photoperiod

## Abstract

Introducing plants in the design of biophilic indoor environments is fundamental for improving human health, well-being, and performance. Previous studies showed that the phenotype of the model plant *Arabidopsis thaliana* grown under LED-sourced CoeLux^®^ lighting systems was characterized by low biomass production rates, a small leaf area, and a low lamina-to-petiole length ratio, suggesting the onset of a strong shade avoidance syndrome. Therefore, it is essential to identify new strategies to improve plant growth under these peculiar light conditions. In the present work, we investigated the effects of two growing media (i.e., low-fertility soil and soil-less substrate), solid and liquid fertilizers, manure, biochar, perlite, mirror reflection of light, and a 24 h photoperiod on *A. thaliana* plants growing under CoeLux^®^ lighting systems at a light intensity of 30 μmol m^−2^s^−1^. We found that the biochar soil amendment to low-fertility soil increases both the above-ground plant biomass and leaf area. Furthermore, the application of a mirror behind the plants and a continuous photoperiod improves not only the biomass and the leaf area but also the lamina-to-petiole length ratio. The combination of different beneficial treatments can further boost plant growth in the low-intensity light environment characterizing the CoeLux^®^ biophilic lighting systems.

## 1. Introduction

**Figure 1:**
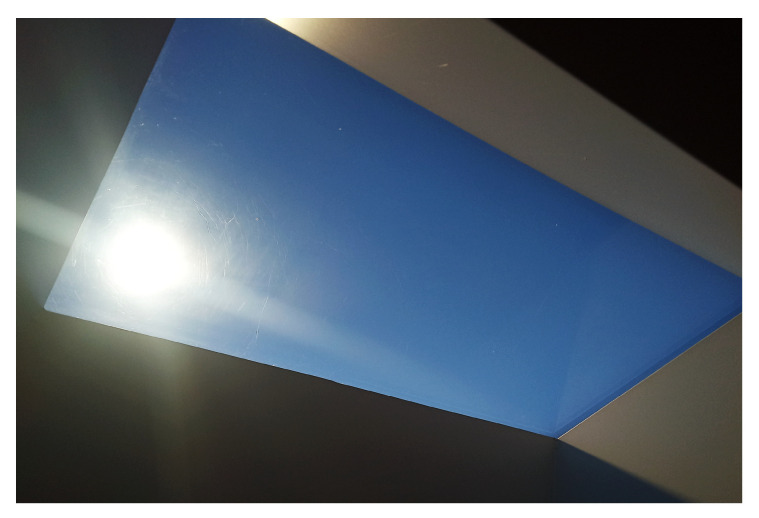
The visual appearance of the CoeLux^®^ lighting systems.

Biophilic design strategies can greatly improve human health, well-being, and performance in indoor environments [1]. A variety of direct or indirect experiences of nature can be used [2], and among them plants and sunlight often play a pivotal role [3]. It has been demonstrated that integrating plants into offices can have significant positive effects on productivity, attention, and creativity as perceived by the occupants [1], enhancing comfort and satisfaction [4] while concomitantly reducing nervousness and anxiety [3]. The use of natural sunlight rather than artificial light can further boost these positive effects [4]. However, direct access to natural sunlight may not be possible in every design situation, such as in underground or specific medical environments. In these contexts, the use of lighting systems able to simulate natural sunlight can help to enhance such biophilic approaches. A recently developed LED-sourced lighting system, named CoeLux^®^, is able to reproduce the visual effect of the sun in a blue sky (Figure 1), providing a real impression of natural sunlight together with all of its proprieties [5,6]. This peculiar room lighting is perceived as more natural, pleasant, and attractive, and it was proposed that it might generate positive long-term psychophysiological effects on human beings, as well as real sunlight [7]. 

In previous studies, we have investigated the impact of the CoeLux^®^ lighting systems on plant growth using the model plant *Arabidopsis thaliana* [8,9] and the aromatic plant species *Mentha x piperita* and *Ocimum basilicum* [10]. The *A. thaliana* phenotype was characterized by low biomass production, a small leaf area, and a low lamina-to-petiole length ratio, while aromatic plants showed low biomass production, large leaf areas, and low leaf mass per area production over control plants grown with high-pressure sodium (HPS) lighting. The data suggested the onset of a strong shade avoidance syndrome (SAS) due to both the spectral composition and low light intensity characterizing the CoeLux^®^ light type. In this context, the study of technical solutions, such as amendments and fertilization enhancing soil chemical properties and light treatments to increase the light intensity, may function as tools for integrating the poor characteristics of the light and improving plant growth in indoor biophilic environments irradiated by the CoeLux^®^ lighting system.

Soil fertility refers to the ability of soil to sustain crop productivity, providing all essential nutrients in a balanced and available form under conditions favorable for plant growth [11]. Low-fertility soils usually have inadequate nutrient supply levels for most agricultural plants and low water retention capacity. In particular, Alisols consist of acidic soils (pH 4.9) that allow the cultivation only of shallow-rooting and acid-tolerant species, which suffer from drought stress in the dry season. When limed and fertilized, crops grown on Alisols may benefit from their considerable cation exchange capacity and good water-holding capacity [12]. On the opposite side, peat-based soil-less substrate growing media are characterized by many favorable characteristics that make them ideal for plant growth, among them a large water-holding capacity (WHC), high air capacity at 100% WHC, low bulk density, and absence of weed seeds and pathogens [13]. However, even if peat-based soil-less substrates may represent optimal growing media for plant growth, peat use for horticultural practices is far from environmentally sustainable, as peat excavation is associated with a consistent ecological footprint and is strongly discouraged by the EU directives [14]. Wet peatlands are fragile ecosystems with important functions such as biodiversity conservation, water purification, and climate regulation [15], as they stock 20% of all global soil carbon despite covering only 3% of the world’s land area [16]. To diminish the human C footprint and preserve biodiversity, it is important to reduce peat use for growing medium production and restore peatlands to re-establish their long-term C sequestration function [17]. In this context, biochar could provide a valid alternative to peat in growing medium formulations [18], as it holds many desirable characteristics that are appreciated in peat. Biochar is the solid by-product of biomass pyrolysis in an oxygen-depleted atmosphere [19] that can be obtained from a wide range of raw materials, including wood chips, plant residues, and algae [20]. This material is characterized by high stability and a porous structure that provides a high specific surface area [21], the key feature that makes biochar such an appreciated amendment. The application of biochar to soil is also hypothesized to increase the bioavailable water, build soil organic matter, enhance nutrient cycling, lower the bulk density, act as a liming agent, and reduce the leaching of pesticides and nutrients to groundwater [22]. Furthermore, biochar represents a stable form of carbon, as its half-life in soil is over 1000 years [22], providing an intriguing potential for carbon storage strategies when used as a soil amendment [23]. Biochar application to low-fertility soils seems to be a good practice to improve soil fertility by retaining nutrients and enhancing the nutrient bioavailability, consequently enhancing crop productivity [24].

In this study, we investigated the effects of growing media, fertilizers, amendments, and light treatments on *A. thaliana* plants growing under CoeLux^®^ lighting systems at a light intensity compatible with a possible end-user requirement. We conceived 7 different treatments and tested them on two different growing media, namely a low-fertility soil (LFS) collected in marginal lands and a commercial soil-less substrate (SLS) composed of peat, compost, and sand. Furthermore, the treatments that showed positive effects on plant growth were tested together to assess the effects of different combinations of multiple treatments. The LFS was chosen to demonstrate that this type of soil, which is usually considered unsuitable for plant production, can be amended with biochar to provide an optimal growing media and a valid alternative to peat-based SLSs characterized by a high environmental impact. Three morphological traits were assessed on *Arabidopsis thaliana* plants subjected to each treatment, namely the biomass production, projected rosette area (PRA), and lamina-to-petiole length ratio (L/P). We observed weaker growth in *A. thaliana* plants grown in the low-fertility soil when compared to plants grown in the commercial growing medium containing sand, peat, and hummus. Furthermore, we observed improvements in biomass production and PRA in plants subjected to biochar amendment in low-fertility soil. Lastly, we observed pronounced improvements of all three morphological traits in treatments involving an increase in light intensity via the reflection of light with a mirror or a 24 h light photoperiod.

## 2. Results

### 2.1. Characteristics of the Growing Media

The main chemical and physical properties of the different growing media and treatments are summarized in Table 1.

In the control treatments, a lower pH was observed in the low-fertility soil (LFS) growing media with respect to the soil-less substrate (SLS) growing media. Within the LFS growing media, treatment with perlite (Pe), biochar (B), solid fertilizer (SF), or liquid fertilizer (LF) significantly increased the pH, while the manure (Ma) treatment reduced the pH. The highest pH values were observed with the biochar amendment treatment (6.46). Within the SLS growing media, the treatment with Pe or B did not affect the growing media pH, while the treatment with Ma, SF, or LF significantly decreased the growing media pH. All treatments showed significantly higher values in the SLS with respect to the LFS growing media, with the only exception of the biochar treatment, where no differences between the two growing media were observed.

The available water content (AWC) showed no differences between the two growing medium control treatments. Furthermore, no differences were observed between the different treatments within the same growing media. A higher AWC was observed in the SLS growing media treated with B, SF, or LF with respect to the same treatments applied to the LFS growing media.

The cation exchange capacity (CEC) was shown to be higher in the SLS with respect to the LFS growing media. No statistically significant differences were observed between the different treatments within the same growing media, with the only exception of the biochar-treated LFS, which showed the highest values within the LFS growing media. All treatments showed significantly higher values in the SLS with respect to the LFS growing media, with the only exception of the biochar treatment, where no differences between the two growing media were observed.

The electrical conductivity (EC) showed no differences between the two growing medium control treatments. Within the LFS growing media, the treatments with Pe, B, and SF showed no statistically significant differences, while the Ma and LF treatments significantly increased the EC. Within the SLS growing media, no statistically significant differences were observed between the different treatments and the CTR. All treatments showed no statistically significant differences between the two growing media analyzed.

The total carbon (C_tot_) was shown to be significantly higher in the SLS with respect to the LFS growing media. Within the LFS growing media, the treatment with Pe, B, Ma, and SF significantly increased the C_tot_, while the treatment with LF showed no statistically significant differences. Within the SLS growing media, all treatments showed lower C_tot_ values with respect to the CTR, with the only exception being the B treatment, which showed significantly higher values. All treatments showed significantly higher values in the SLS with respect to the LFS growing media.

The organic carbon (C_org_) showed no differences between the two growing medium control treatments. Within the LFS growing media, no statistically significant differences were observed between the different treatments, with the only exception of the LF treatment, which showed lower values. Within the SLS growing media, the treatments with SF and LF showed no statistically significant differences, while the Pe, B, and Ma treatments significantly increased the C_org_. A higher C_org_ was observed in the SLS growing media treated with B, Ma, or SF with respect to the same treatments applied to the LFS growing media.

The total nitrogen (N_tot_) was shown to be significantly higher in the SLS with respect to the LFS growing media. Within the LFS growing media, the treatments with B and SF showed no statistically significant differences, while the Pe, Ma, and LF treatments significantly increased the N_tot_. Within the SLS growing media, the treatments with Pe, Ma, and SF showed no statistically significant differences, while the B and Ma treatments significantly increased the N_tot_. The LF treatment showed significantly lower values with respect to the CTR. All treatments showed significantly higher values in the SLS with respect to the LFS growing media, with the only exception being the LF treatment, where no differences between the two growing media were observed.

The organic nitrogen (N_org_) showed no differences between the two growing medium control treatments. Furthermore, no differences were observed between the different treatments within the same growing media. A higher N_org_ was observed in the SLS growing media treated with Ma with respect to the same treatment applied to the LFS growing media.

The total phosphorus (P_tot_) was shown to be significantly higher in the SLS with respect to the LFS growing media. Within the LFS growing media, all treatments showed higher P_tot_ values with respect to the CTR, with the only exception being the Pe treatment, which showed no statistically significant differences. Within the SLS growing media, the treatments with Pe and Ma showed no statistically significant differences, while the SF and LF treatments significantly increased the P_tot_. The B treatment showed significantly lower values with respect to the CTR. All treatments showed significantly higher values in the SLS with respect to the LFS growing media.

The available phosphorus (P_av_) was shown to be significantly higher in the SLS with respect to the LFS growing media. Within the LFS growing media, the treatments with Pe, Ma, and SF showed no statistically significant differences, while the B and LF treatments significantly increased the P_av_. Within the SLS growing media, the treatments with Pe and SF showed no statistically significant differences, while the B and Ma treatments showed significantly lower values with respect to the CTR. The LF treatment showed significantly higher values with respect to the CTR. All treatments showed significantly higher values in the SLS with respect to the LFS growing media, with the only exception of the B treatment, where the lowest values were observed in the SLS growing media.

The total potassium (K_tot_) was shown to be significantly higher in the SLS with respect to the LFS growing media. Within the LFS growing media, all treatments showed a higher K_tot_ with respect to the CTR. Within the SLS growing media, all treatments showed a higher K_tot_ with respect to the CTR, with the only exception of the Pe treatment, which showed no statistically significant differences. All treatments showed significantly higher values in the SLS with respect to the LFS growing media, with the only exception of the SF treatment, where the lowest values were observed in the SLS growing media.

The available potassium (K_av_) showed no differences between the two growing medium control treatments. Within the LFS growing media, all treatments showed a higher K_tot_ with respect to the CTR, with the only exception of the B treatment, which showed no statistically significant differences. Within the SLS growing media, all treatments showed a higher K_tot_ with respect to the CTR, with the only exception of the Pe treatment, which showed no statistically significant differences. A higher K_av_ was observed in the SLS growing media treated with Ma or SF with respect to the same treatments applied to the LFS growing media, while lower values were observed with the Pe treatment.

### 2.2. Morphological Traits of the Plants

**Figure 2:**
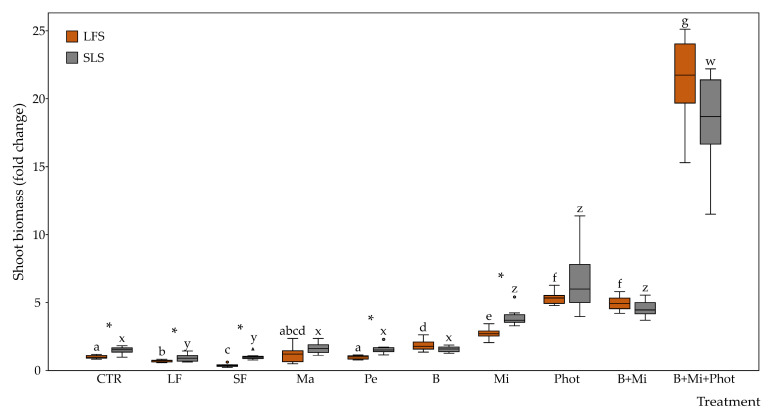
Fold changes in shoot biomass between plants grown under the CoeLux^®^ lighting systems with different treatments. Control plants grown in LFS with no additional treatments were used as the reference group. Vertical boxes represent approximately 50% of the observations (*n* = 12 biological repeats) and lines extending from each box are the upper and lower 25% values of the distribution. Circles and triangles represent respectively outliers and extreme outliers. Asterisks indicate statistically significant differences (*p* < 0.05) between plants grown with LFS or SLS, while letters indicate differences (*p* < 0.05) between the different treatments within the same growing media. Abbreviations: LFS: low-fertility soil; SLS: soil-less substrate; CTR: control; LF: liquid fertilizer treatment; SF: solid fertilizer treatment; Ma: manure treatment; Pe: perlite treatment; B: biochar treatment; Mi: mirror treatment; Phot: photoperiod treatment.

The shoot biomass values measured in plants grown in low-fertility soil (LFS) showed no differences between plants grown in the growing media alone (CTR) and plants grown in growing media mixed with perlite (Pe) or manure (Ma). On the contrary, plants grown with the addition of liquid fertilizer (LF) or solid fertilizer (SF) showed biomass decreases of 0.7- and 0.3-fold, respectively, while plants grown LFS added with biochar (B), irradiated by the additional mirror light (Mi) or under a continuous photoperiod (Phot), showed biomass increases of 1.8-, 2.7-, or 5.3-fold the CTR value, respectively. Combining the B and mirror (Mi) treatments, the plants showed a 5.0-fold biomass increase, while when combining the B, Mi, and Phot treatments, the plants showed a 21.3-fold biomass increase (Figure 2). 

The shoot biomass values measured in plants grown in the soil-less substrate (SLS) showed no differences between the SLS CTR treatment (1.5-fold the LFS CTR) and plants subjected to the Ma, Pe, or B treatment. On the contrary, the plants subjected to the LF or SF treatments showed biomass decreases ranging from 1.5-fold to 0.9-fold, while the plants subjected to the Mi or Phot treatments showed biomass increases ranging from 1.5-fold to 4.2- or 6.4-fold. When combining the B and Mi treatments, the plants showed a 4.6-fold biomass increase, while when combining the B, Mi, and Phot treatments, the plants showed an 18.6-fold biomass increase (Figure 2).

When the two growing media were compared, the plants grown in the SLS showed a 1.5-fold biomass increase with respect to the CTR plants grown in LFS. Plant biomass increases between the SLS and the LFS growing media were observed also in the LF, SF, and Pe treatments, while no differences were observed in the Ma, B, Mi, and Phot treatments. When multiple treatments were applied, namely B + Mi or B + Mi + Phot, no statistically significant differences were observed between the two different growing media (Figure 2). 

**Figure 3:**
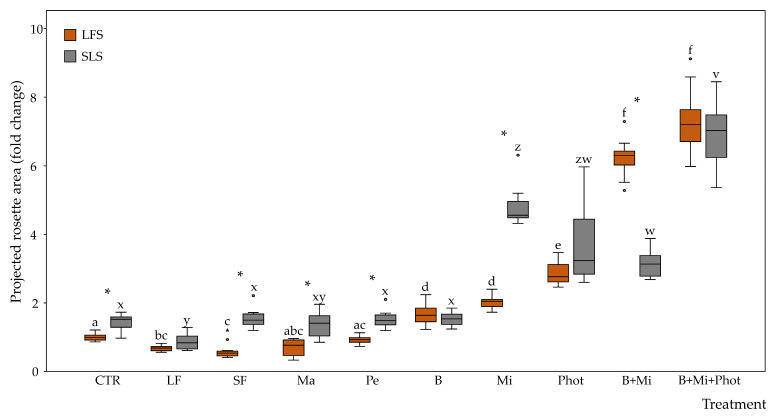
Fold changes in projected rosette areas between plants grown under the CoeLux^®^ lighting systems with different treatments. Control plants grown in LFS with no additional treatments were used as the reference group. Vertical boxes represent approximately 50% of the observations (*n* = 12 biological repeats) and lines extending from each box are the upper and lower 25% values of the distribution. Circles and triangles represent respectively outliers and extreme outliers. Asterisks indicate statistically significant differences (*p* < 0.05) between plants grown with LFS or SLS, while letters indicate differences (*p* < 0.05) between the different treatments within the same growing media. Abbreviations: LFS: low-fertility soil; SLS: soil-less substrate; CTR: control; LF: liquid fertilizer treatment; SF: solid fertilizer treatment; Ma: manure treatment; Pe: perlite treatment; B: biochar treatment; Mi: mirror treatment; Phot: photoperiod treatment.

The projected rosette area (PRA) measured in plants grown in LFS showed no differences between the CTR and the Ma or Pe treatments. On the contrary, the plants subjected to the LF or SF showed PRA decreases of 0.7- and 0.5-fold, respectively, compared to the CTR, while the plants subjected to the B, Mi, and Phot treatments showed PRA increases of 1.7-, 2.0-, and 2.9-fold, respectively. When combining the B and Mi treatments, the plants showed a 6.2-fold PRA increase, while when combining the B, Mi, and Phot treatments, the plants showed a 7.3-fold PRA increase (Figure 3).

The PRA measured in the plants grown in the soil-less substrate (SLS) showed no differences between the SLS CTR treatment (1.4-fold the LFS CTR) and the plants subjected to the SF, Ma, P, or B treatments. On the contrary, the plants subjected to the LF treatment showed biomass decreases ranging from 1.4-fold to 0.9-fold, while the plants subjected to the Mi and Phot treatments showed biomass increases ranging from 1.4-fold to 4.8- and 3.6-fold. When combining the B and Mi treatments, the plants showed a 3.1-fold PRA increase, while when combining the B, Mi, and Phot treatments, the plants showed a 6.9-fold PRA increase (Figure 3).

When the two growing media were compared, the plants grown in the SLS showed a 1.4-fold increase in the PRA with respect to the CTR plants grown in LFS. PRA increases between the SLS and the LFS growing media were observed also in the SF, Ma, Pe, and Mi treatments, while no differences were observed in the LF, B and Phot treatments. On the contrary, when both the biochar and mirror treatments were applied, the plants grown in the LFS showed higher PRA values with respect to plants grown in the SLS growing media. When the B + Mi + Phot multiple treatments were applied, no statistically significant differences were observed between the two different growing media (Figure 3).

**Figure 4:**
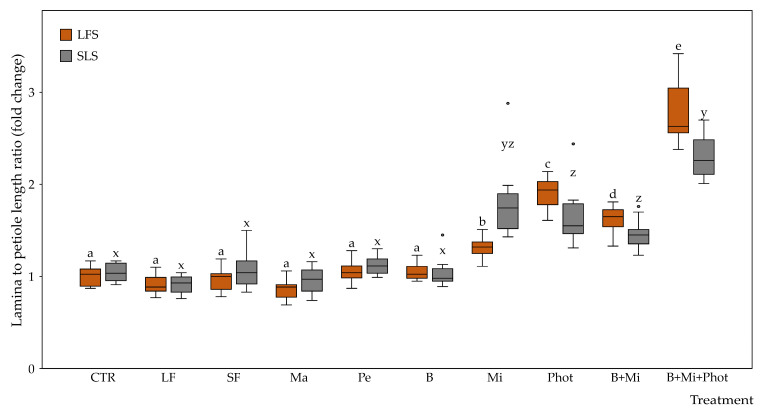
Fold changes in lamina-to-petiole length ratios between plants grown under the CoeLux^®^ lighting systems with different treatments. Control plants grown in LFS with no additional treatments were used as the reference group. Vertical boxes represent approximately 50% of the observations (*n* = 12 biological repeats) and lines extending from each box are the upper and lower 25% values of the distribution. Circles represent outliers. Letters indicate differences (*p* < 0.05) between the different treatments within the same growing media. Abbreviations: LFS: low-fertility soil; SLS: soil-less substrate; CTR: control; LF: liquid fertilizer treatment; SF: solid fertilizer treatment; Ma: manure treatment; Pe: perlite treatment; B: biochar treatment; Mi: mirror treatment; Phot: photoperiod treatment.

The lamina-to-petiole length ratio (L/P) values measured in plants grown in LFS showed no differences between the CTR and the LF, SF, Ma, Pe, or B treatments. On the contrary, the plants subjected to the Mi or Phot treatments showed L/P increases of up to 1.3- and 1.9-fold compared to the CTR. When combining the B and Mi treatments, the plants showed a 1.6-fold L/P increase, while when combining the B, Mi, and Phot treatments, the plants showed a 2.8-fold L/P increase (Figure 4).

The L/P values measured in plants grown in the soil-less substrate (SLS) showed no differences between the SLS CTR treatment (1.0-fold the LFS CTR) and the plants subjected to the LF, SF, Ma, Pe, or B treatments. On the contrary, the plants subjected to the Mi or Phot treatments showed L/P increases of up to 1.8- and 1.7-fold compared to the CTR. Combining the B and Mi treatments, the plants showed a 1.4-fold L/P increase, while combining the B, Mi, and Phot treatments, the plants showed a 2.3-fold L/P increase (Figure 4).

When the two growing media were compared, the plants grown in the SLS growing media showed no statistically significant differences in the L/P with respect to the CTR plants grown in the LFS growing media. Furthermore, no statistically significant differences were observed for all treatments applied, including the B + Mi and B + Mi + Phot multiple treatments.

## 3. Discussion

**Figure 5:**
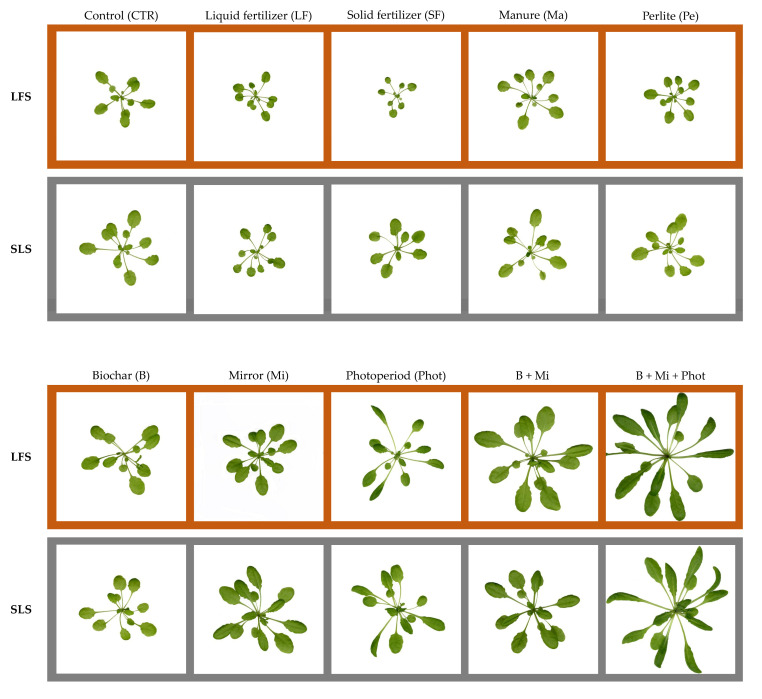
Comparison of representative rosette phenotypes of *Arabidopsis thaliana* plants grown in low-fertility soil (LFS: orange) or soil-less substrate (SLS: grey) and subjected to the diverse treatments. White squares have a 10 cm side.

In the present work, three morphological traits were assessed on *A. thaliana* plants growing under CoeLux^®^ lighting systems in response to treatments involving two different growing media and the application of fertilization, amendment, or light treatments. The plants showed specific responses to each treatment tested (Figure 5). When only the two different growing media were considered, the control plants grown in the soil-less substrate (SLS) showed a higher biomass production rate and a larger projected rosette area (PRA) when compared to the control plants grown in low-fertility soil (LFS), which explains why peat-based SLSs are so commonly used in horticultural practices. These growing media are characterized by many favorable characteristics that make them ideal for plant growth, among them a large water-holding capacity (WHC), large air capacity at 100% WHC, low bulk density, and absence of weed seeds and pathogens [13]. In addition, the SLS growing media used in our study showed higher cation exchange capacity (CEC), total carbon (C_tot_), total nitrogen (N_tot_), total phosphorous (P_tot_), available phosphorous (P_av_), and total potassium (K_tot_) values when compared to the LFS-grown media (Table 1).

### 3.1. Specific Amendments Can Improve Plant Growth under Biophilic Lighting

Among the two treatments with the amendments, perlite (Pe) and biochar (B), very different results were obtained. The Pe treatment showed no statistically significant differences with respect to the control plants in both growing media and for all three morphological traits measured. On the contrary, the plants subjected to the B treatment showed increases in both biomass and PRA when grown in LFS, while no differences were observed when grown in SLS. We hypothesized that the LFS benefitted more than SLS from the positive effects of the biochar amendment, as SLS already holds many of the favorable characteristics provided by the biochar amendment, such as a low bulk density and high nutrient supply level. Additionally, the soil analysis summarized in Table 1 showed increases in pH, CEC, C_tot_, P_tot_, P_av_, and K_tot_ in the LFS amended with 20% *v*/*v* orchard pruning biochar. These results suggest that the biochar application could improve the LFS growing media’s quality to a level comparable to that observed in the SLS growing media. This observation is of particular importance when the environmental impacts of peat-based SLSs are considered, as the use of peat for the formulation of growing media is far from environmentally sustainable. As with the many publications that report positive applications of biochar in soil [12] and horticultural growing media [25], our results demonstrated the suitability of this amendment solution for improving plant growth under biophilic LED-sourced lighting while maintaining a low ecological footprint.

### 3.2. Fertilization Showed No Positive Effects on Plants Growing under Limited Light Conditions

Three fertilization treatments were tested using different commercial products, namely a liquid fertilizer (LF), a solid fertilizer (SF), and bovine and equine manure (Ma). No biomass or PRA increase was observed in plants subjected to one of these treatments; rather, biomass and PRA decreases were observed in plants subjected to the LF or SF treatments in both growing media. Under normal light conditions, the use of fertilizers is a common strategy for increased plant biomass and leaf area; however, our data demonstrate that in limited light conditions, this strategy does not work and could even lead to adverse effects. Similar results were obtained by Deng et al., who tested different levels of fertilization and shading on *Cyclocarea paliurus*, observing the lower biomass production in the treatment with the higher shading and fertilization levels [26]. In contrast, plants subjected to the same shading treatment but with lower fertilization rates showed better performances [26]. Furthermore, Grubb et al. reported little or no ability to respond to additional nutrients in woody species subjected to moderate to deep shade levels [27]. Considering the low light intensity available under the biophilic CoeLux^®^ lighting systems, the use of fertilization treatments is not a suggested strategy to improve plant growth under these lighting conditions.

### 3.3. Mirror Reflection and a Continuous Light Photoperiod Can Boost Plant Growth under Biophilic Lighting

Among the different treatments tested, the plants subjected to the mirror (Mi) and photoperiod (Phot) treatments showed overall better results, as consistent biomass and PRA increases were observed with both growing media. In the Mi treatment, a +56% light intensity increase was enough to boost the plant biomass production up to 2.7-fold that of the CTR in the LFS growing media and 3.9-fold that of the CTR in the SLS growing media, while in the Phot treatment, the enhancement from 14 h/d light to 24 h/d light boosted the plant biomass production by up to 5.3-fold that of the CTR in the LFS growing media and 6.4-fold that of the CTR in the SLS growing media. Furthermore, under both treatments, significant increases were also observed in the lamina-to-petiole length ratio (L/P) values. A low L/P is considered a hallmark response in *A. thaliana* plants growing under unfavorable light conditions [28]. None of the plants subjected to the other treatments showed an increase in the L/P ratio, suggesting that increasing the light quantity irradiating the plants is the only efficient way to improve this morphological trait. These observations are not surprising, as plants depend upon light for their survival [29]. It was demonstrated that in conditions of low light intensity, the rate of photosynthesis is almost directly proportional to the light intensity if other factors are not limiting. Consequently, the amount of biomass produced increases with the increase in light intensity up to a certain maximum, after which the biomass decreases once again [30]. Furthermore, it was already observed that the duration of light in a 24 h photoperiod can dramatically affect *A. thaliana*’s biomass production, with a significant biomass increase with the increase in light hours [31]. Thus, the use of technical solutions to raise the light quantity reaching the plants, both in terms of the light intensity and light duration, is strongly advised when growing plants under biophilic lighting systems or in conditions of low light intensity. 

Nevertheless, the use of a mirror offers a further advantage to plants growing under CoeLux^®^-like lighting systems, as it provides the plants with a more uniform light source. Indeed, the light emitted from the CoeLux^®^ systems reaches the plants at a fixed angle of 45°, causing the plants to bend in the direction of the light, favored by the increased etiolation caused by the SAS. The application of a mirror behind the plant provides light from a different angle, favoring plant growth with a more standard shape. Furthermore, both the Mi and Phot treatments can be easily coupled with specific growing media or other treatments (e.g., biochar soil amendment), which could offer further growth enhancements.

### 3.4. Combined Treatments Can Lead to Even Better Growth Performance

We also tested two combinations of multiple treatments by applying both the B and Mi (B + Mi) treatments or the B, Mi, and Phot (B + Mi + Phot) treatments together. With the B + Mi treatment applied to the LFS growing media, we observed a 5-fold increase in biomass production with respect to the CTR, a higher increase than observed when applying the same treatments singularly to LFS. On the contrary, in the SLS growing media, no significant differences were observed between the B + Mi treatment and the Mi treatment alone, showing that the addition of biochar to the SLS produced no beneficial effects. A similar pattern was also observed for the PRA and L/P traits, as the addition of biochar to the Mi treatment resulted in higher PRA and L/P values in the LFS growing media and not in the SLS growing media. Our data are in accordance with the observations in a previous study on *Fagus sylvatica* [32]. When this species was growing with a higher light intensity and in soil with a higher nutrient content, Minotta et al. observed higher shoot biomass and leaf area values. This observation is further supported by the B + Mi + Phot treatment, where the average daily light integral increase (DLI) up to 4.1 mol m^−2^d^−1^ resulted in a 21.3-fold biomass increase in the LFS growing media and an 18.6-fold biomass increase in the SLS growing media. The enhanced light quantity reaching the plants also resulted in the highest L/P ratio, emphasizing the enhanced plant health. These data suggest that the positive effects of single treatments can be joined together to obtain better results than a single treatment alone. Furthermore, we observed that the interplay between different positive treatments (B + Mi + Phot) leads to biomass and PRA values exceeding the value corresponding to the sum of the different treatments’ effects analyzed singularly. These findings highlight the existence of a synergistic effect of biochar, mirror reflection, and a continuous photoperiod, which is still controversial in the published literature. For example, Seehausen et al. found no synergistic effects when using biochar and compost together on *Abutilon theophrasti* and *Salix purpurea* plant growth [33]. On the contrary, Miyagi et al. found a synergistic effect of monochromic LEDs combined with high CO_2_ and nutrient levels on the development of *Lactuca sativa* [34]. Furthermore, several papers showed a synergistic effect of plant-growth-promoting rhizobacteria strains [35,36] alone or with arbuscular mycorrhizal fungi against abiotic stress [37].

## 4. Materials and Methods

### 4.1. Plant Materials and Growth Conditions 

*Arabidopsis thaliana* Col-8 wild-type (N60000) seeds were purchased from the Eurasian Arabidopsis Stock Centre (NASC). The seeds were stratified at 4 °C for 5 days on 1% agar gel and subsequently transferred to pot flats (Araflats; Arasystem; Ghent/Belgium) composed of 51 individual pot cavities with a 5 cm diameter and an 80 mL volume, filled with two different types of sterilized growing media. The plants were grown at a temperature as close as possible to 22 °C, with air humidity values ranging between 50% and 70% and a photoperiod of 14 h. The light was provided by two CoeLux^®^ 45HC lighting systems sourced by full-spectrum white LEDs with a color temperature of 6500 K. The plants were grown at a height of 1 m from the floor level of the growth room and 2.05 m from the lighting systems, a position that we believe will be used by the end-users of the CoeLux^®^ systems. Within this setup, the plants were reached by light intensity rates ranging between 25 and 36 μmol m^−2^s^−1^, with an incident angle of 45° and an average daily light integral (DLI) of 1.5 mol m^−2^d^−1^.

### 4.2. Growing Media and Imposed Treatments 

**Figure 6:**
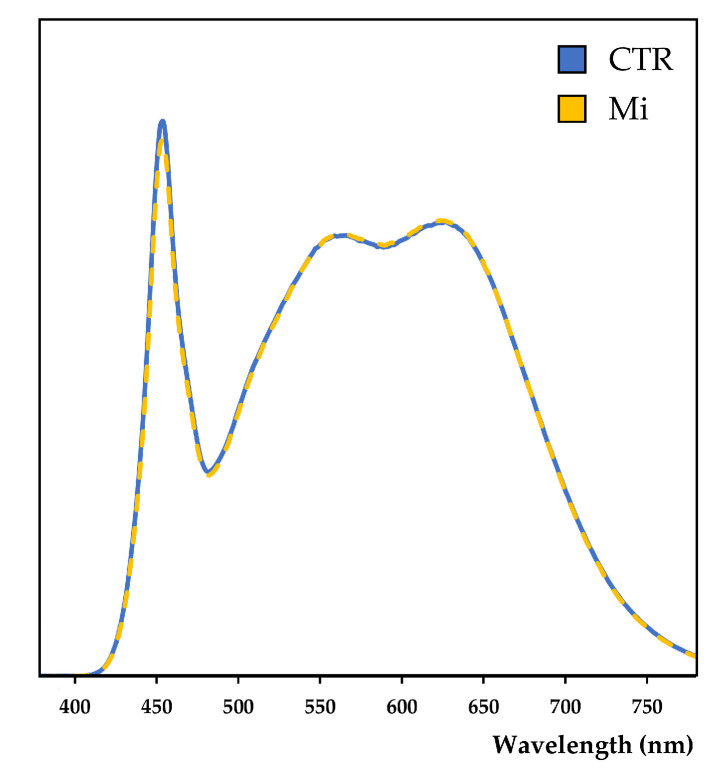
Spectral curves measured within the control (blue) and mirror (yellow) treatments between 380 nm and 780 nm. The spectral curves represent the means of 6 different measurements (*n* = 6). CTR: control treatment; Mi: mirror treatment.

Two different growing media, (i) a low-fertility soil (LFS) collected at the University of Insubria campus (45°47′52.6″ N 8°51′17.5″ E; 392 m a.s.l.), classified as Alisol by the World Reference Base for Soil Resources (WRB), and (ii) a commercial soil-less substrate (SLS) mixture composed of acidic *Sphagnum* peat, green compost, and siliceous sand (2:1:1 *w*/*w*), were tested alone (CTR = control) and in combination with the following treatments:

➢ Liquid fertilizer (LF): 1 mL of liquid fertilizer (Concime per piante verdi—Compo—Italy), with a NPK ratio of 7.5:3:6, was diluted in 1 L of tap water and supplied weekly to the plants’ tray. The first application was provided 10 days after sowing;
➢ Solid fertilizer (SF): 0.1 g of solid fertilizer (Blu concime universale—Compo—Italy) with a NPK ratio of 12:12:17 was applied directly on the growing media surface 10 days after sowing;
➢ Manure (Ma): Commercial bovine and equine manure pellets (Stallatico micro pellettato—Vigorplant^®^—Italy) were crushed with a mortar and pestle and sieved with a mesh of 2 mm. A quantity of 10 mL of manure was thoroughly mixed with 990 mL of growing media to obtain a 1% *v*/*v* concentration of manure;
➢ Perlite (Pe): 500 mL of agricultural perlite (Agrilit^®^ 3—Perlite Italiana srl; pH 6.5–7.5) was thoroughly mixed with 500 mL of growing media to obtain a 50% *v*/*v* concentration;
➢ Biochar (B): The biochar used in this study was produced by Romagna Carbone s.n.c. (Italy) from orchard pruning biomass through a slow pyrolysis process with an average residence time of 3 h at 500 °C [12]. The raw biochar was crushed with a mortar and pestle and sieved with a mesh of 2 mm. A quantity of 200 mL of biochar was thoroughly mixed with 800 mL of growing media to obtain a 20% *v*/*v* concentration of biochar;
➢ Mirror (Mi): A mirror was placed behind the tray to reflect part of the artificial sunlight that would not reach the plants. With this setup, the light intensity ranged between 40 and 55 μmol m^−2^s^−1^, with an average DLI of 2.4 mol m^−2^d^−1^. Spectrum measurements every 1 nm in the range between 380 and 780 nm were taken on a horizontal white reflector using the Spectraval 1511 instrument (JETI Technische Instrumente GmbH—Germany), both with and without the mirror’s presence, to assess that no spectral variations were introduced by the mirror’s application (Figure 6). To allow a comparison, photon counts measurements were normalized on the luminance of the respective spectrum;
➢ Photoperiod (Phot): A 24 h light photoperiod was applied with an average DLI of 2.6 mol m^−2^d^−1^;
➢ Biochar and mirror (B + Mi): Both B and Mi treatments were applied;
➢ Biochar, mirror, and photoperiod (B + Mi + Phot): The B, Mi, and Phot treatments were applied. The average DLI was 4.1 mol m^−2^d^−1^.

### 4.3. Analysis of Growing Media

The pH and electrical conductivity (EC) of the growing media were measured in three replicates on 1:5 (*v*/*v*) growing media/water extracts using a portable meter (PC7, Hydro Tech, Rosolini). The cation exchange capacity (CEC) was determined using an NH_4_OAc method. The available water content (AWC) was obtained with a pressure plate apparatus operating between −0.33 (FC-field capacity) and −15 MPa (WP-wilting point) in the laboratory on disturbed samples [38] by desorbing the saturated cores at different pressure steps. The soil samples were placed on a porous pressure plate apparatus (ceramic) and then installed in the pressure chambers. The cores were initially saturated overnight from the bottom under a small head of water. The water content at each pressure step was calculated from the volume of outflow between the pressure steps, the final water content, and the weight of the oven-dried soil. The available water content was calculated as the difference between the WP (wilting point) and FC (field capacity). 

The total nitrogen (N_tot_), total carbon (C_tot_), and organic carbon (C_org_) values were determined via dry combustion using a CHN elemental analyzer (Carlo Erba Instruments, mod 1500 series 2). In the case of C_org_, the combustion was carried out after the complete removal of inorganic C with acid. The available nitrogen (N_av_) was determined using a modified Kjeldahl procedure using Devarda’s alloy [39] as a reducing agent to convert the NO_3_ and NO_2_ into NH_4_^+^, with subsequent Kjeldahl digestion. The total phosphorus (P_tot_) content was determined via spectrophotometry (UV-1601 Shimadzu) according to the test method described by Bowman [40]. The available phosphorus (P_av_) was extracted by a NaHCO_3_ solution at pH 8.5 and evaluated by spectrophotometry according to the Olsen method [41]. The K_tot_ was determined according to EPA method 3052 [42] using an ICP-OES spectrophotometer (Varian Inc., Vista MPX, Palo Alto, CA, USA). The K_av_ was quantified by extraction with BaCl_2_-triethanolamine followed by ICP-OES spectrophotometry.

### 4.4. Plant Analysis

A total of 12 plants for each treatment were sampled 37 days after sowing. The whole shoot of each plant was scanned at 800 dpi with the Epson Expression 12000XL instrument (Figure 5) and then oven-dried at 70 °C until reaching a constant weight. The scanned images were processed with WinRhizo (Regent Instrument) to measure the leaf area in the form of the projected rosette area (PRA) and with ImageJ (NIH, Bethesda, MD, USA) to measure the lamina and petiole lengths of the two bigger leaves of each plant. The lamina to petiole length ratio (L/P) was then calculated. SPSS Statistics 25 (IBM) was used to run the post hoc Dunnett’s test for multiple comparisons. Statistically significant differences (*p* < 0.05) between the means were marked with the letters a, b, c, d, e, f, and g for the LFS treatments, with the letters x, y, z, w, and v being used for the SLS treatments and a black asterisk for comparisons between the two different growing media. All data were normalized to the control LFS treatment to highlight fold change differences. 

## 5. Conclusions

This research showed that the selection of a proper growing medium and proper amendment or fertilization treatments is of central importance to improving plant growth in the peculiar light environment found under the CoeLux^®^ lighting system. We demonstrated that fertilization treatments are not effective for boosting plant growth in conditions of limited lighting and can even produce detrimental outcomes. On the contrary, the biochar-based amendment was shown to be an effective tool to increase both the biomass and leaf area. However, the most effective solutions we identified consisted of enhancing the light quantity reaching the plant by placing a mirror right behind the plants to increase the light intensity irradiating them or increasing the photoperiod to a 24 h light photoperiod. In addition to the biomass and leaf area increases, these solutions allowed the mitigation of shade avoidance syndrome (SAS) symptoms such as a low lamina-to-petiole length ratio and leaf orientation toward the light source. Furthermore, the coupling of the best solutions allowed us to obtain the best overall results, greatly increasing the biomass production and leaf area and simultaneously reducing the SAS symptoms. 

Our future research will focus on testing further treatments involving light availability to plants, biochar amendment proportions, and the coupling of different treatments and growing media to identify favorable combinations. Furthermore, the application of the best treatments detected to diverse plant species could provide further evidence about the effectiveness of this approach.

## Figures and Tables

**Table 1:** Chemical and physical characteristics of the different growing media and treatments used in this study. Each value represents the mean of 3 replicates (*n* = 3) ± 95% confidence interval. Letters a, b, c, d, e indicate significant differences (*p* < 0.05) between the different treatments within the low-fertility soil growing media, while letters x, y, z, w, v, u indicate significant differences (*p* < 0.05) within the soil-less substrate growing media. Asterisks indicate significant differences (*p* < 0.05) between the two growing media within the same treatment.

**Growing Media**	**Treatment**	**pH**			**AWC** **(m^3^ m^−3^)**			**CEC** **(cmol kg^−1^)**			**EC** **(dS m^−1^)**		
Low-fertility soil	CTR	5.32	±0.05	a *	0.088	±0.004	a	12.58	±0.13	a *	1.23	±0.08	a
	Pe	6.03	±0.05	b *	0.089	±0.009	a	12.43	±0.40	a *	1.30	±0.30	ab
	B	6.46	±0.09	c	0.085	±0.006	a *	14.40	±0.20	b	1.77	±0.20	ab
	Ma	5.04	±0.08	d *	0.114	±0.014	a	11.73	±0.80	a *	1.73	±0.10	b
	SF	5.77	±0.02	e *	0.097	±0.010	a *	11.97	±0.40	a *	1.83	±0.20	ab
	LF	5.92	±0.03	b *	0.100	±0.006	a *	12.93	±0.30	a *	1.83	±0.10	b
Soil-less substrate	CTR	6.52	±0.03	x *	0.167	±0.032	x	16.50	±0.30	x *	1.43	±0.30	xy
	Pe	6.67	±0.06	x *	0.157	±0.051	x	16.57	±0.10	x *	1.37	±0.20	xy
	B	6.62	±0.10	xy	0.174	±0.007	x *	17.23	±1.00	x	1.37	±0.10	x
	Ma	6.28	±0.07	zw *	0.150	±0.062	x	16.40	±0.50	x *	1.63	±0.10	y
	SF	6.36	±0.03	yz *	0.186	±0.021	x *	17.23	±0.70	x *	1.77	±0.30	xy
	LF	6.21	±0.03	w *	0.171	±0.011	x *	17.33	±1.00	x *	1.77	±0.10	y
**Growing media**	**Treatment**	**C_tot_** **(%)**			**C_org_** **(%)**			**N_tot_** **(%)**			**N_org_** **(%)**		
Low-fertility soil	CTR	1.80	±0.01	a *	1.66	±0.37	a	0.16	±0.00	ab *	0.15	±0.03	ab
	Pe	2.02	±0.01	b *	2.48	±0.69	ab	0.18	±0.00	b *	0.12	±0.00	a
	B	3.23	±0.01	c *	2.44	±0.49	a *	0.18	±0.00	ab *	0.15	±0.01	b
	Ma	2.09	±0.02	d *	2.63	±0.76	a *	0.20	±0.00	c *	0.14	±0.01	ab *
	SF	1.86	±0.01	e *	1.33	±0.07	ab *	0.18	±0.00	a *	0.13	±0.02	ab
	LF	1.96	±0.07	abde *	0.54	±0.42	b	0.19	±0.00	c	0.14	±0.06	ab
Soil-less substrate	CTR	9.06	±0.01	x *	2.39	±0.14	x	0.23	±0.01	xz *	0.21	±0.03	xy
	Pe	8.62	±0.03	y *	2.90	±0.06	y	0.27	±0.01	xyz *	0.25	±0.07	xy
	B	11.87	±0.07	z *	9.45	±0.21	z *	0.27	±0.00	y *	0.23	±0.06	xy
	Ma	8.32	±0.01	w *	6.60	±0.09	w *	0.25	±0.01	z *	0.23	±0.01	x *
	SF	8.21	±0.02	v *	2.46	±0.21	xy *	0.27	±0.00	yz *	0.22	±0.04	xy
	LF	6.22	±0.00	u *	1.60	±0.50	xy	0.19	±0.01	w	0.16	±0.01	y
**Growing media**	**Treatment**	**P_tot_** **(ppm)**			**P_av_** **(ppm)**			**K_tot_** **(ppm)**			**K_av_** **(ppm)**		
Low-fertility soil	CTR	574.5	±0.53	a *	0.38	±0.01	a *	1129.3	±5.70	a *	19.4	±0.08	a
	Pe	579.8	±2.82	a *	0.46	±0.03	a *	1267.7	±0.47	b *	22.5	±0.25	b *
	B	706.2	±0.59	b *	0.56	±0.01	b *	1283.8	±23.67	b *	23.3	±1.62	abc
	Ma	605.5	±0.89	c *	0.41	±0.01	a *	1777.7	±3.32	c *	25.7	±0.44	c *
	SF	590.7	±0.54	d *	0.41	±0.01	a *	1893.8	±5.09	d *	292.6	±3.41	d *
	LF	603.3	±2.75	c *	1.23	±0.07	c *	1778.8	±0.61	c *	314.5	±10.45	d
Soil-less substrate	CTR	878.8	±0.66	x *	0.98	±0.01	x *	1677.5	±3.13	x *	20.3	±0.32	x
	Pe	868.4	±8.44	x *	0.96	±0.09	xz *	1679.0	±0.02	x *	19.6	±0.44	x *
	B	577.4	±6.02	y *	0.39	±0.01	y *	1787.1	±3.91	y *	26.0	±1.15	y
	Ma	880.9	±1.11	x *	0.90	±0.01	z *	2739.7	±0.99	z *	103.4	±2.22	z *
	SF	998.1	±2.54	z *	1.03	±0.04	xz *	1784.9	±1.35	y *	314.3	±0.89	w *
	LF	1005.7	±2.11	w *	1.89	±0.04	w *	2949.3	±27.90	w *	320.9	±8.17	w

Abbreviations: AWC: available water content; CEC: cation exchange capacity; EC: electrical conductivity; tot: total; org: organic; av: available; CTR: control; P: perlite treatment; B: biochar treatment; Ma: manure treatment; SF: solid fertilizer treatment; LF: liquid fertilizer treatment.

## Data Availability

The data presented in this study are available on request from the corresponding author.
